# Polarity Determinants in Dendritic Spine Development and Plasticity

**DOI:** 10.1155/2016/3145019

**Published:** 2015-12-29

**Authors:** Huaye Zhang

**Affiliations:** Department of Neuroscience and Cell Biology, Rutgers Robert Wood Johnson Medical School, Piscataway, NJ 08854, USA

## Abstract

The asymmetric distribution of various proteins and RNAs is essential for all stages of animal development, and establishment and maintenance of this cellular polarity are regulated by a group of conserved polarity determinants. Studies over the last 10 years highlight important functions for polarity proteins, including apical-basal polarity and planar cell polarity regulators, in dendritic spine development and plasticity. Remarkably, many of the conserved polarity machineries function in similar manners in the context of spine development as they do in epithelial morphogenesis. Interestingly, some polarity proteins also utilize neuronal-specific mechanisms. Although many questions remain unanswered in our understanding of how polarity proteins regulate spine development and plasticity, current and future research will undoubtedly shed more light on how this conserved group of proteins orchestrates different pathways to shape the neuronal circuitry.

## 1. Introduction

Neurons are probably the most polarized/compartmentalized cell type in the human body. Their polarity establishment starts with the specification of dendrites and axons. Further compartmentalization occurs during the formation of dendritic spines, which receive most of the excitatory synaptic inputs in the brain. Thus, the formation and maintenance of dendritic spines can be seen as a localized form of polarity establishment, where separation and maintenance of different membrane and cytoplasmic domains are needed. This makes proteins regulating cellular polarity ideally suited to function in dendritic spine development. Indeed, recent studies from a number of laboratories highlight key roles for different classes of polarity proteins in dendritic spine development and plasticity. In this review, I will summarize recent advances in studying the role of cell polarity regulators, including apical-basal polarity and planar polarity determinants, in dendritic spine development and plasticity, and discuss possible future avenues of investigation.

## 2. The Spine Cytoskeleton

The actin and microtubule cytoskeleton provides the structural basis for cell polarity in most cell types. For example, asymmetric actin polymerization allows a migrating cell to polarize and extend lamellipodia in the direction of movement. In addition, polarized vesicular trafficking along microtubules is essential for the establishment and maintenance of apical versus basolateral domains in epithelial cells [1]. Similarly, dendritic spines depend on the unique organization of the cytoskeleton to maintain their polarized morphology. Dendritic spines are highly actin-rich structures that extend from the microtubule-rich dendritic shaft. Spines typically consist of an enlarged spine head containing a dense network of short branched actin filaments. The spine head is connected to the main dendritic shaft through the spine neck, which contains both long linear and short branched actin filaments [2–4]. Although actin constitutes the main cytoskeletal element of dendritic spines, dynamic microtubules do enter spines, a process that is regulated by neuronal activity [5, 6]. This activity-dependent microtubule invasion is important for synaptic plasticity [7–9]. Thus, the dynamic actin and microtubule cytoskeleton is important for the morphogenesis and plasticity of dendritic spines. Not surprisingly, many of the upstream polarity regulators target the cytoskeleton to regulate spine growth, maturation, and function, as will be discussed in the following sections.

## 3. Partitioning-Defective (Par) Proteins

**Figure 1:**
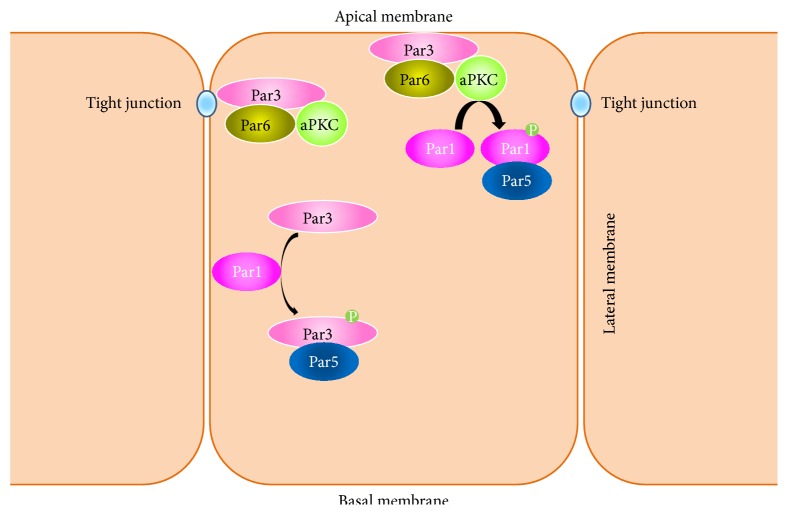
Par polarity proteins maintain their polarized distribution through a mutual exclusion mechanism. In epithelial cells, the Par3/Par6/aPKC complex is localized to the apical membrane while Par1 is localized to the basolateral membrane. Par1 is phosphorylated by aPKC, which leads to the binding of Par1 with Par5, a 14-3-3 protein. This interaction will lead to the inhibition of Par1 membrane binding and kinase activity. In this way, Par1 is excluded from the membrane domain occupied by the Par3/Par6/aPKC complex. Conversely, Par1 can phosphorylate Par3, which leads to Par5/14-3-3 binding and triggers the release of Par3 from the cell membrane, thus preventing the Par3/6 complex from localizing to the lateral membrane.

The partitioning-defective (Par) proteins play an essential role in various contexts of polarity establishment, including embryogenesis, directional motility, epithelial morphogenesis, and axon specification [10]. These proteins were initially discovered in the* C. elegans *zygote, where mutations in the* par* genes cause defects in partitioning of the zygote into asymmetric daughter cells [11]. The Par proteins (except for Par2) are conserved from worms to mammals. Par1 and Par4 are Ser/Thr kinases. Par3 and Par6 are PDZ domain-containing scaffolding/adaptor proteins. Par5 is a member of the 14-3-3 family of proteins, which binds to phosphorylated Ser/Thr residues [12]. Par proteins can have distinct distribution patterns. For example, in the developing zygote, Par3 and Par6, which form a complex with atypical PKC (aPKC), are localized to the anterior pole while Par1 is localized to the posterior pole. In epithelial cells, the Par3/Par6/aPKC complex is localized apically, while Par1 is localized basolaterally. This polarized distribution is partially achieved by the two complexes mutually excluding each other from their respective domains [13]. Par1 is phosphorylated by aPKC, which leads to the binding of Par1 with Par5. This interaction will lead to the inhibition of Par1 membrane binding and kinase activity. In this way, Par1 is excluded from the membrane domain occupied by the Par3/Par6/aPKC complex [14, 15]. Conversely, Par1 can phosphorylate Par3, which leads to Par5/14-3-3 binding and triggers the release of Par3 from the cell membrane [16], thus preventing the Par3/6 complex from localizing to the lateral membrane (Figure 1). This mutual exclusion mechanism helps cells establish and maintain polarity by compartmentalizing signaling processes in a spatially specific manner.

The highly compartmentalized nature of neurons and their dendritic spines makes Par proteins ideal candidates to function in spine morphogenesis and plasticity. Indeed, the Par3/Par6/aPKC complex was found to play an important role in dendritic spine morphogenesis in hippocampal neurons. Depletion of Par3 results in immature spines that are filopodial- and lamellipodial-like [17]. This phenotype is mediated by the guanine nucleotide exchange factor TIAM1, which activates the small GTPase Rac1. TIAM1 interacts directly with the C-terminus of Par3 [18, 19]. Further experiments show that Par3 functions by spatially restricting Rac activation to dendritic spines through targeting TIAM1. Since Rac is a key regulator of actin dynamics, it was proposed that Par3 and TIAM1 locally modulate the actin cytoskeleton, which is important for proper spine development. In the absence of Par3, TIAM1 becomes mislocalized causing aberrant activation of Rac, which disrupts normal spine morphogenesis [17]. Recently, the adhesion G-protein coupled receptor (GPCR) brain-specific angiogenesis inhibitor 1 (BAI1) was found to be the upstream regulator of the Par3/TIAM1 complex [20]. BAI1 interacts with the Par3/TIAM1 complex and targets it to dendritic spines. In the absence of BAI1, the Par3/TIAM1 complex is mislocalized, and Rac activation is lost in dendritic spines. These recent results elegantly demonstrate for the first time a cell surface receptor that targets and regulates the Par polarity complex at the postsynapse. It also positions the Par3/TIAM1 complex in a key position to link a synaptic adhesion receptor to local modulation of actin dynamics.

While Par3 functions through TIAM1 and Rac in spine morphogenesis [17], the Par6/aPKC complex was also found to play a distinct role in spine development. Overexpression of Par6 or enzymatic activation of aPKC promotes spine development, while depletion of Par6 or inhibiting aPKC disrupts spine morphogenesis. Unexpectedly, the Par6/aPKC complex was found to function through p190 RhoGAP and the small GTPase RhoA. Overexpression of Par6 inhibits RhoA activation while knockdown of Par6 elevates RhoA activity [21]. Since prolonged activation of RhoA negatively regulates spine development [22, 23], the Par6/aPKC complex promotes spine development by keeping RhoA activity low in dendritic spines. It is interesting to note that in* Drosophila* the Par3/Par6/aPKC complex regulates glutamatergic synapse formation at the neuromuscular junction (NMJ) by modulating actin and microtubule dynamics [24, 25]. Moreover, the localization of Par3 and Par6 to the NMJ is dependent on aPKC kinase activity [24, 25], and the retention of Par3 at the NMJ depends on its dephosphorylation by the lipid and protein phosphatase PTEN [24]. Whether similar mechanisms are involved in the mammalian dendritic spines remains to be determined.

**Figure 2:**
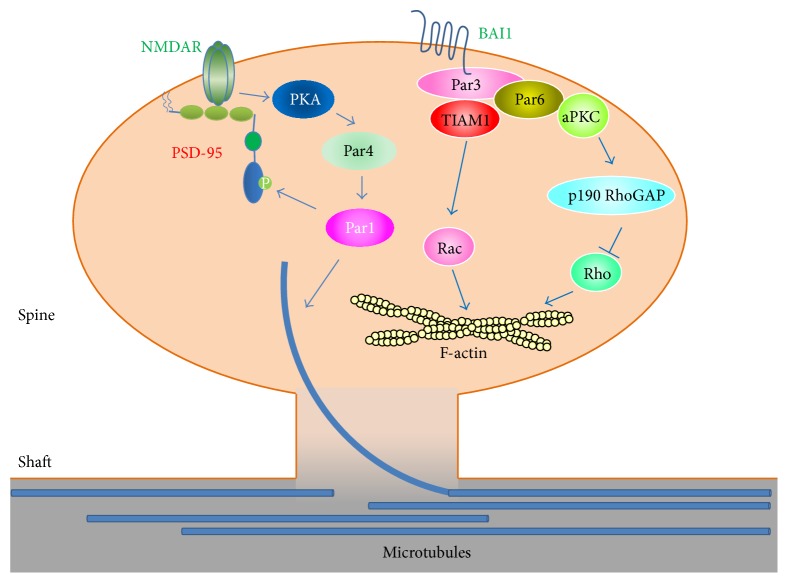
Par polarity proteins in dendritic spines. Members of the partitioning-defective (Par) polarity proteins regulate dendritic spine development through different pathways. Par1 functions downstream of NMDA receptors (NMDAR) to regulate dynamic microtubules and to phosphorylate PSD-95. Par3 functions downstream of the BAI1 receptor and targets TIAM1 to modulate Rac activity. Par6 and aPKC function through p190 RhoGAP to inhibit RhoA. Both Rac and Rho are central regulators of actin dynamics.

As mentioned above, in developing zygotes and epithelia, the Par3/Par6/aPKC complex antagonizes the function of another polarity protein, Ser/Thr kinase Par1, also known as the Microtubule Affinity Regulating Kinase (MARK). The mammalian Par1/MARK was originally discovered as a family of kinases that phosphorylates microtubule-associated proteins (MAPs), such as MAP2 and tau, leading to the disassembly of microtubules [26]. There are four members of the mammalian Par1/MARK family, including Par1c/MARK1, Par1b/MARK2, Par1a/MARK3, and Par1d/MARK4. A number of other substrates have since been identified, including doublecortin [27, 28], histone deacetylase 7 (HDAC7) [29], plakophilin 2 [30], Cdc25 [31], and Par3 [16, 32]. In rat hippocampal neurons, depletion of Par1b/MARK2 inhibits dendritic spine maturation, resulting in elongated filopodia-like protrusions. Live imaging studies revealed that in Par1b depleted neurons microtubule growth is reduced. Further, it was found that the microtubule plus end binding protein p140Cap showed reduced accumulation in dendritic spines when Par1b was depleted [33]. Together these studies suggest that Par1 promotes dendritic spine development through modulating microtubule dynamics. Interestingly, in* Drosophila*, the Par1/MARK homolog dPar1 phosphorylates discs large (Dlg) and regulates neuromuscular junction formation [34]. This phosphorylation mechanism is conserved as the mammalian Par1/MARK phosphorylates the Dlg homolog PSD-95 on the conserved Ser561 site. Phosphorylation of this site is important for the function of Par1 in dendritic spine morphogenesis, as a phosphomimetic mutant of PSD-95 can rescue the spine formation defects in hippocampal neurons expressing kinase-dead Par1 [35]. In addition, Par1/MARK was found to function downstream of NMDA receptors through a mechanism that depends on PKA and another member of the Par proteins, Par4, also known as LKB1 [36]. Together, these studies show that Par1 is important for spine development through regulating both microtubule dynamics and the synaptic scaffolding protein PSD-95. It will be interesting to examine whether Par1 participates in NMDA receptor-dependent synaptic plasticity and whether the known antagonistic effects of the Par4/Par1 and Par3/Par6 complexes play any role in spine development (Figure 2).

## 4. The Septin GTPases

Septins are cytoskeletal proteins that regulate cell polarity by forming filamentous structures underneath the plasma membrane to function as diffusion barriers. They belong to the GTPase family that binds to and hydrolyzes GTP into GDP. There are 13 mammalian septin genes, many of which exist in multiple isoforms [37]. Different septins interact with each other to form heterooligomeric complexes. These oligomers then assemble end-to-end to form filamentous structures. Septin filaments can be straight, curved, or circular and function as scaffolds and/or diffusion barriers [38]. For example, in the budding yeast* Saccharomyces cerevisiae*, where these proteins were initially discovered over 40 years ago, septins form a ring around the neck between mother and bud [37, 38]. More recent studies show that this septin diffusion barrier is important for the asymmetric segregation of age during yeast budding. Aging factor such as circular DNA is retained in the mother cell by a septin-dependent lateral diffusion barrier. This ensures that age is reset in the newborn bud so species propagation can be achieved [39].

**Figure 3:**
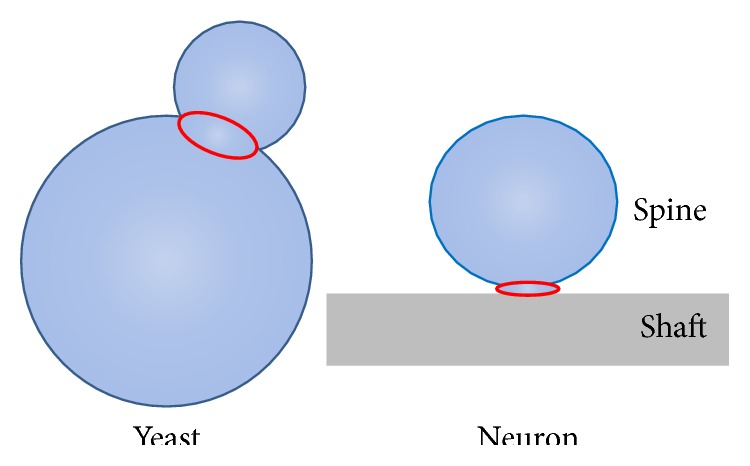
Septin diffusion barriers in yeast and spines. Septins form filamentous structures that constitute diffusion barriers in the yeast bud neck. Similarly, septin diffusion barriers have been found in the spine neck.

Given the geometrical similarities between a yeast bud and a dendritic spine, different groups hypothesized that septins may form a ring around the spine neck to limit diffusion in and out of dendritic spines, thus biochemically compartmentalizing the spine (Figure 3). Indeed it was known that a fraction of dendritic spines are diffusionally isolated [40]; however the molecular identity of this barrier was not clear at the time. In 2007, two groups discovered that septins are indeed present at the spine neck and play an important role in dendritic spine morphogenesis. Both groups independently found that septin 7 (Sept7) is localized to the base of dendritic filopodia, branch points, and the base of dendritic spines. Overexpression of Sept7 increases dendritic branching and protrusion density [41], while depletion of Sept7 results in reduced dendritic arborization and immature, elongated spines [41, 42], suggesting that Sept7 is important for spine maturation.

While the localization of Sept7 to spine neck indicates a role in barrier function, this was not experimentally demonstrated until a recent study by the Choquet group [43]. They measured diffusion of the GluA2 receptor, bulk membrane, and cytoplasmic proteins across the spine neck, using fluorescence recovery after photobleaching (FRAP) imaging. Diffusion of GluA2 and membrane-bound mRFP is slower in spines containing the septin barrier, while diffusion of cytoplasmic mRFP is not affected [43]. This suggests that Sept7 regulates the lateral diffusion of membrane proteins in and out of spines, which is in line with known septin functions in other organisms. It is intriguing to speculate that septins contribute to the heterogeneity of dendritic spines by forming a barrier on certain spine necks but not others. Further research is needed to elucidate how septin-containing spines and septin-free spines differ in their physiological functions.

## 5. Planar Cell Polarity Proteins

Planar cell polarity (PCP) is a phenomenon in which coordinated orientation of cells and their appendages, such as stereocilia or hair, occurs within the plane of the epithelial sheet. Thus in the case of PCP, asymmetry is established at the tissue level rather than the cellular level. Genetic studies in* Drosophila* have revealed conserved PCP proteins such as Frizzled (Fz), Dishevelled (Dvl), and Van Gogh (Vang). Studies in the mammalian cochlea have identified additional PCP factors including Vangl2 (a mammalian homologue of the* Drosophila* Vang) and Scrb1 (mammalian homologue of the* Drosophila* Scribble) [44]. From a basic cell biological perspective, the core function of PCP proteins is similar to other polarity proteins, which is to compartmentalize the membrane, except that the compartmentalization occurs on the anterior-posterior body axis instead of the apical-basal axis. Thus, it is perhaps not surprising that several of the PCP proteins are also found to be important for dendritic spine morphogenesis.

### 5.1. Scribble

Scribble (Scrib) is a large scaffolding protein containing 16 leucine-rich repeats (LRR) on the N-terminus followed by four PDZ domains. It was originally identified in* Drosophila* as a determinant of apical-basolateral polarity [45] and a tumor suppressor [46]. Scrib localizes to the basolateral domain of epithelial cells and promotes basolateral membrane identity together with its binding partners lethal giant larvae (Lgl) and discs large (Dlg). Depletion of Scrib disrupts E-cadherin mediated adhesion in Madin-Darby Canine Kidney epithelial cells [47, 48]. In mammalian cochlear hair cells, a mutation in the Scrib gene causes defects in PCP as reflected by disrupted orientation of stereociliary bundles of hair cells [49]. Furthermore, Scrib genetically and physically interacts with the PCP core protein Vang and functions as its effector during PCP establishment in* Drosophila* [50]. Thus Scrib is a determinant of both apical-basal polarity and planar polarity.

In* Drosophila*, Scrib regulates the architecture of the presynaptic terminal. Scrib mutant flies show fewer synaptic vesicles in the active zone and more in the reserve pool, resulting in defects in short-term synaptic plasticity [51]. In mammals, this presynaptic effect of Scrib is believed to be downstream of *β*-catenin [52]. On the postsynaptic side, Scrib recruits the neuronal nitric oxide synthase 1 adaptor protein (NOS1AP) to the G-protein coupled receptor interacting protein 1 (GIT1)/*β*-p21-activated kinase- (PAK-) interacting exchange factor (*β*-PIX)/PAK complex to regulate dendritic spine morphogenesis. As the GIT1/*β*-PIX complex functions to regulate PAK activity through Rac [53, 54], the Scrib-NOS1AP complex also regulates spine morphogenesis through influencing Rac activity [55]. Indeed Scrib mutant mice show increased Rac activation [56]. Furthermore, these mutant mice show impaired synaptic transmission and plasticity in the hippocampus. Overall dendritic spine density is reduced in Scrib mutant mice; however individual spines are enlarged [56]. Together these studies suggest that Scrib functions through Rac to regulate dendritic spine development and plasticity.

### 5.2. The Wnt/Fz/Dvl Pathway and Vangl

Wnts are a family of secreted proteins that are important for many aspects of tissue development. Wnt proteins function through the seven-transmembrane Frizzled receptor (Fz) and the cytoplasmic adaptor protein Disheveled (Dvl). There are two main branches of the Wnt signaling pathway. The canonical Wnt pathway involves downstream phosphorylation of *β*-catenin and regulation of gene transcription. The noncanonical Wnt PCP pathway involves regulation of RhoA and actomyosin contractility [57]. During animal development, the Wnt PCP pathway regulates key processes such as convergent extension and neural tube closure [58]. The Wnt pathway is also crucial for multiple cellular processes during brain development, including proliferation and differentiation of neuronal precursors [59], neuronal migration [60], and axon guidance [61]. More recent studies show that Wnt signaling promotes dendritic spine formation in hippocampal neurons [62]. Several different Wnts, including Wnt2, Wnt5a, and Wnt7a, have been shown to increase dendritic spine density [63–65]. Wnt5a increases synaptic transmission [64] and clustering of PSD-95 [66], and Wnt7a increases excitatory, but not inhibitory, synaptic transmission through Dvl1 and the calcium-calmodulin dependent kinase II (CaMKII) [65]. The specific receptors mediating these effects include Fz5, which may act both pre- and postsynaptically [67]. Other Fz receptors involved may include Fz1 and Fz3, both of which are highly localized to synaptic sites [68]. It will be interesting to examine the involvement of other Wnt receptors, including the receptor tyrosine kinase Ryk and receptor tyrosine kinase-like orphan receptor 2 (ROR2). Indeed a recent study shows that depletion of ROR2 inhibits dendritic spine maturation [69].

**Figure 4:**
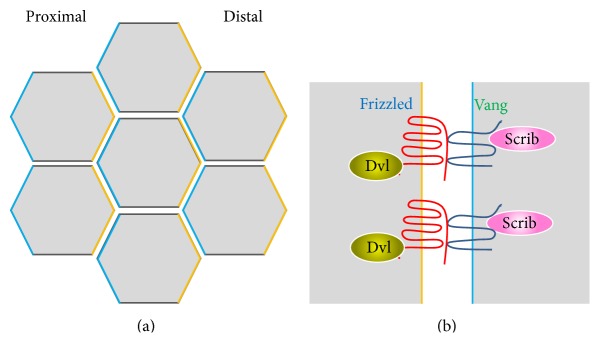
Asymmetric localization of core planar cell polarity (PCP) proteins in the fly wing. (a) Cells can polarize within the plane of the epithelial sheet, a phenomenon called planar cell polarity. (b) The core PCP proteins Frizzled and Vang form transcellular interactions and are distributed asymmetrically along the epithelial plane. Vang is concentrated in the proximal membrane while Frizzled is localized to the distal membrane.

**Figure 5:**
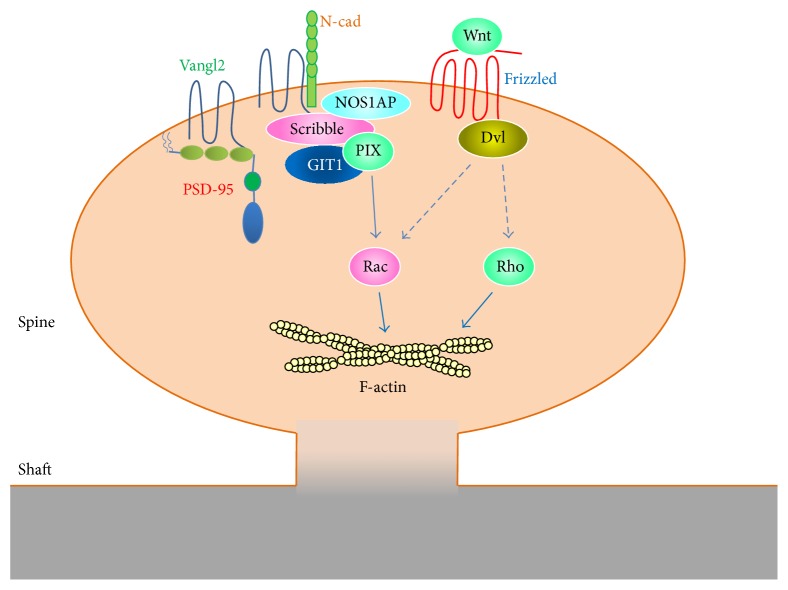
Planar cell polarity proteins in dendritic spines. Planar cell polarity (PCP) proteins play important roles in dendritic spine development. Wnt signals via the Frizzled receptor and Disheveled (Dvl) to regulate spine morphogenesis, possibly through the Rho GTPases. The PCP protein Vangl2 interacts directly with PSD-95 and N-cadherin and regulates N-cadherin endocytosis. Vangl2 also interacts with Scribble, which forms a complex with NOS1AP, GIT1, and PIX to regulate Rac activity in dendritic spines. Dashed arrows represent pathways that have been established in other cell types but have not been directly demonstrated in dendritic spines.

The* Drosophila* Vang and its mammalian homologue Vangl are tetramembrane spanning proteins that function as core components of the PCP pathway. In the* Drosophila* wing epithelia, Fz and Vang segregate into distinct domains [70]. Fz concentrates on the distal edges of cells while Vang localizes to the proximal edges. How this spatial segregation is achieved is unclear and several different models have been proposed [71]; however the direct transcellular interaction between Fz and Vang is likely involved [72, 73] (Figure 4). In vertebrates, there are two Vangl genes, Vangl1 and Vangl2. Vangl2 is highly expressed in neuronal tissues and regulates various aspects of brain development including neurulation [74, 75], neuronal migration [76, 77], and growth cone guidance [78]. Recent studies show that Vangl2 is also important for dendritic spine development. Vangl2 forms a direct interaction through its C-terminal PDZ-binding motif with PSD-95 on the third PDZ domain [79]. In addition, Vangl2 directly interacts with N-cadherin and enhances its internalization [80]. In hippocampal neurons depleted of Vangl2, both dendritic branching and spine density are reduced [81]. Formation of synapses is also reduced as shown by the decreased clustering of pre- and postsynaptic markers [80]. These studies show that Vangl2 is important for dendritic spine development. It will be interesting to determine how interactions between different PCP proteins contribute to spine development and plasticity (Figure 5).

## 6. Crosstalk between Polarity Proteins

The interplay within and between different groups of polarity proteins has been most extensively examined in epithelial cells of* Drosophila* and mammals. As described above, the reciprocal exclusions of the Par1-Par3/Par6/aPKC complexes and the Fz-Vang complexes are important for the establishment of apical-basal and planar cell polarity, respectively. However how interactions within different groups of polarity proteins contribute to dendritic spine development and function is largely unknown. Since the interplay between polarity proteins is important for establishing different cellular domains in nonneuronal cells, it is intriguing to speculate that these reciprocal interactions are involved in establishing different spine domains or subdomains. Recent studies using superresolution microscopy have revealed interesting microdomain organizations within dendritic spines [82]. It will be interesting to see whether the organization of these microdomains depends on the balancing acts of the polarity complexes.

Crosstalk between different groups of polarity proteins also occurs. As described above, Scribble interacts with both apical-basal polarity determinants like Lgl and PCP determinants like Vang. Interestingly, recent studies show that the apical-basal polarity determinants Par3/Par6/aPKC can become planar polarized [83, 84], which leads to different fates of the daughter cells [84]. This indicates crosstalk between the Par complex and the PCP machinery. Indeed the Wnt/Dvl pathway has been shown to regulate the Par complex through the interaction between Dvl and aPKC [85]. Finally, Par4/LKB1 and Par1/MARK can regulate the basolateral localization of Scribble [86]. How these crosstalks are involved in dendritic spine development and function remains to be determined.

Interestingly, many of these polarity determinants target the actin and microtubule cytoskeleton to regulate spine development and plasticity. For example, the Par complex, Scribble, and the Wnt/Dvl complex all target the Rho family GTPases, which are core regulators of the actin cytoskeleton. Rho GTPases have also been shown to modulate microtubule dynamics [87]. Moreover, Par1 and Wnt/Dvl are known regulators of microtubule dynamics [88, 89]. Further studies will shed light on how signals from diverse groups of polarity determinants converge on the cytoskeleton to modulate dendritic spine development and function.

## 7. Conclusions

The establishment of cell polarity is essential at all stages of animal development, as segregation of different cellular domains is key to the physiological functions of all cell types. Studies from traditional model systems, such as* S. cerevisiae*,* C. elegans*, and* D. melanogaster*, have provided significant insight into the mechanisms by which a conserved group of polarity proteins, including apical-basal polarity proteins and planer polarity proteins, functions in different contexts of polarity establishment. Recent studies in mammalian neurons have highlighted the remarkable diversity of functions for this conserved group of cell polarity proteins. Evolution has bestowed novel roles upon these polarity regulators in the development of dendritic spines, which is a more complex level of neuronal compartmentalization that occurs primarily in vertebrates. While great progress has been made in understanding the function of this important group of proteins in spine development, many questions remain. For example, dendritic spines are heterogeneous in both their morphology and function. Do polarity proteins regulate this heterogeneity? Some polarity proteins show segregated distribution in epithelial cells. Do they distribute to different spine subdomains in neurons? If so, how does this contribute to synaptic functions? Recent advances in imaging techniques, including superresolution imaging, will help address some of these questions. Future research will pave the way to understanding of how these conserved polarity proteins help shape the synaptic connections and how they contribute to cognitive functions of the brain.
